# Curcumin Regulated the Homeostasis of Memory T Cell and Ameliorated Dextran Sulfate Sodium-Induced Experimental Colitis

**DOI:** 10.3389/fphar.2020.630244

**Published:** 2021-02-01

**Authors:** You-Bao Zhong, Zeng-Ping Kang, Bu-Gao Zhou, Hai-Yan Wang, Jian Long, Wen Zhou, Hai-Mei Zhao, Duan-Yong Liu

**Affiliations:** ^1^ Department of Postgraduate, Jiangxi University of Traditional Chinese Medicine, Nanchang, China; ^2^ College of Traditional Chinese Medicine, Jiangxi University of Traditional Chinese Medicine, Nanchang, China; ^3^ Laboratory Animal Research Center for Science and Technology, Jiangxi University of Traditional Chinese Medicine, Nanchang, China; ^4^ Formula-Pattern Research Center of Jiangxi University of Traditional Chinese Medicine, Nanchang, China; ^5^ Science and Technology College, Jiangxi University of Traditional Chinese Medicine, Nanchang, China

**Keywords:** curcumin, experimental colitis, JAK1/STAT5 signaling pathway, memory T cells, mechanism of action

## Abstract

Immune memory is protective against reinvasion by pathogens in the homeostatic state, while immune memory disorders can cause autoimmune disease, including inflammatory bowel disease. Curcumin is a natural compound shown to be effective against human inflammatory bowel disease and experimental colitis, but the underlying mechanism is unclear. Here, experimental colitis was induced by dextran sulfate sodium (DSS) in this study. Significant changes in the percentages of naïve, central memory T (TCM), and effector memory (TEM) cells and their CD4^+^ and CD8^+^ subsets were found in the peripheral blood of mice with colitis using flow cytometry. After 7 days of continuous curcumin (100 mg/kg/day) administration, the DSS-induced experimental colitis was effectively relieved, with significant decreases in the ratio of day weight to initial body weight, colonic weight, pathological injury score, levels of proinflammatory cytokines IL-7, IL-15, and IL-21, colonic mucosal ulceration, and amount of inflammatory infiltrate. Importantly, curcumin significantly restored the percentages of naïve, TCM, and TEM cells and their CD4^+^ and CD8^+^ subpopulations. In addition, curcumin significantly inhibited the activation of the JAK1/STAT5 signaling pathway, downregulation of JAK1, STAT5, and p-STAT5 proteins in colon tissue, and upregulation of PIAS1 proteins. These results suggested that curcumin effectively regulated the differentiation of naïve, TCM, and TEM cells in the peripheral blood to alleviate DSS-induced experimental colitis, which might be related to the inhibition of JAK1/STAT5 signaling activity.

## Introduction

Inflammatory bowel disease (IBD) refers to chronic autoimmune diseases of the gastrointestinal tract, including Crohn's disease (CD) and ulcerative colitis (UC) (Hodson, 2016). IBD is a global disease with a high prevalence in developed countries (prevalence >0.3%) (Ungaro et al., 2017) and an accelerating prevalence in newly industrialized countries, especially in Asia (Ng et al., 2018). In addition, IBD has a high recurrence rate and is so difficult to cure to the extent that the World Health Organization has classified it as one of the modern intractable diseases (Jackson and De Cruz, 2019). The advent of IBD has created a serious medical burden on national healthcare finance and has influenced the quality of life of patients. The etiology and pathogenesis of IBD are not fully understood. However, growing evidence indicates that these disorders are the result of the interplay between genetic, environmental, intestinal microflora and immune factors (Xavier and Podolsky, 2007). In particular, T-cell-mediated inflammation is important in experimental colitis and human IBD (Strober et al., 2007; Zundler and Neurath, 2017).

Immune memory dysfunction is a typical feature of IBD onset (Gattinoni et al., 2011). Immune memory is the phenomenon in which the immune system, after initial contact with an antigen that produces a specific immune response, usually responds again when it encounters that antigen. The re-response produced by immune memory is often characterized by rapid, strong, specific elevation and persistence. It can prevent the recurrence of the same disease and play a protective role for the organism, while immune memory disorders can result in the dysregulation of the inflammatory response and local tissue injury (Natoli and Ostuni, 2019). Some studies have shown that memory cells, including central memory T cells (CD45RA^−^CD62L^+^CCR7^+^, TCM) and effector memory T cells (CD45RA^−^CD62L^−^CCR7^-^, TEM), are involved in the onset and course of autoimmune diseases including rheumatoid arthritis and IBD (Belarif et al., 2018; Pardieck et al., 2018). IBD recurrence may be caused by the long-term colonization of colitogenic memory T cells (Kanai et al., 2009; Gattinoni et al., 2011). Excessive colitogenic CD4^+^ central memory T cells (CD4^+^ TCM) are preferentially retained in the bone marrow of mice with colitis, and they circulate between the bone marrow and the lamina propria (Whiteoak et al., 2018). The aforementioned studies suggested that restoring memory T-cell homeostasis is one of the effective measures of IBD therapy.

Importantly, a natural anti-inflammatory compound, curcumin, can effectively relieve chronic IBD by regulating T-cell proliferation and differentiation (Gao et al., 2004; Pang et al., 2018). Curcumin has antioxidant properties; it inhibits the activity of ribonucleotide reductase and DNA polymerases in the cell cycle (Shakeri and Boskabady, 2017), thereby effectively inhibiting lymphocyte proliferation. In addition, curcumin inhibits nuclear factor κB (NF-κB) signal activation and the production of proinflammatory cytokines activated by T cells, including Th1-type cytokines interleukin (IL)-2 and interferon (IFN)-γ, further inhibiting lymphocyte proliferation (Ukil et al., 2003). In CD, Th1 cells are the major drivers of acquired immune-related inflammation (Brand, 2009). Curcumin can block Th1 subpopulation differentiation by inhibiting IL-12 production by macrophages and can promote Th2 subpopulation proliferation and secretion of anti-inflammatory cytokines IL-4 and IL-10 (Zhang et al., 2006). For example, 30 mg/kg curcumin treatment promoted Th2 differentiation and inhibited Th1 proliferation in a rat model of TNBS-induced colitis (Zhang et al., 2006). Curcumin also inhibited the development of Th17, thereby reducing the production of the proinflammatory cytokines IL-6, IL-21, and IL-17 (Bakir et al., 2016). However, whether curcumin has an effect on memory T-cell differentiation and subpopulation homeostasis has not been reported at home or abroad.

In addition, the JAK/STAT signaling pathway has been shown to play an important role in memory T-cell differentiation (Ashrafizadeh et al., 2020). IL-7 promotes the survival of human CD4^+^ effector/memory T cells by activating the JAK/STAT signaling pathway (Chetoui et al., 2010). However, evidence showing that drugs can modulate the differentiation of memory T cells or activate JAK-STAT signaling to treat IBD is scarce. Based on the aforementioned findings, it was hypothesized that curcumin might regulate the differentiation balance of memory T cells to treat IBD. The present study investigated the mechanism underlying curcumin treatment of IBD by observing the number of naïve, TEM, and TCM cells and the expression levels of related proteins in the JAK1/STAT5 signaling pathway in mice with colitis. The data from this study might contribute to a better understanding of the mechanism underlying curcumin treatment of IBD and provide experimental evidence for curcumin as a possible treatment for UC.

## Materials and Methods

### Mice

Male specific pathogen-free (SPF) BALB/c (aged 8–9 weeks, weighing 20–22 g) were purchased from the Hunan Slack Landscape Laboratory Animal Co. Ltd. (Changsha, China) (Animal Certificate Number SCXK (Xiang) 2016-0002). The mice were bred and maintained under SPF conditions, and experiments were conducted following the Institutional Animal Care and Use Committee at the animal facility of Traditional Chinese Medicine (Nanchang, China). The experimental protocols were approved by the Animal Care and Use Committee of Jiangxi University of Traditional Chinese Medicine (identification code: JZLLSC2018-024; date of approval: September 22, 2018).

All mice were acclimatized for 3 days prior to starting the study. Forty mice were randomly and equally divided into 4 groups with 10 mice in each group: normal group (Nor), DSS group (DSS), DSS + Cur group (DSS + Cur), and DSS + mesalazine group (DSS + 5-ASA).

### Induction and Treatment of Experimental Colitis

To induce colitis in mice, male BALB/c mice in the DSS, DSS + Cur group and DSS + 5-ASA group were administered with 3.0% (*w*/*v*) dextran sulfate sodium (batch number: 160110; DSS, 36–50KDs; MP Biomedicals, CA, United States) in drinking water for 7 days. The mice in the normal group received normal drinking water.

Before administration, curcumin (batch number: GR-133-140421; Gangrun Biotechnology, Nanjing, China) was dissolved in 1.5% carboxymethyl cellulose solution at a dose of 100 mg/kg (purity >95% by high performance liquid chromatography (HPLC)). On day 8, the mice in the DSS + Cur and DSS + 5-ASA groups were administered, respectively, curcumin (100 mg/kg) and mesalazine (300 mg/kg; batch number: 130407; Sunflower Pharma, Jiamusi, China) by oral gavage for 7 days; the mice in the normal and DSS groups were treated with equal volume of saline. Throughout the study, all mice were weighed once daily (09:00) and monitored daily for diarrhea, hematochezia, hunched posture, and hair loss.

### Pathological Histology Analysis

The colons were dissected, washed with phosphate-buffered saline (PBS, pH = 7.3), fixed with precooled 4.0% paraformaldehyde (PFA) overnight at 4°C, dehydrated using an alcohol gradient (from 50% to 100%), rendered transparent in xylol, and embedded in paraffin. Then, these samples were cut into 4 μm thickness sections. After deparaffinization and rehydration, the slices were stained with hematoxylin-eosin (H and E) (Solarbio, Beijing, China). Subsequently, the slices were imaged with a biomicroscope (Lecia, Wetzlar, Germany).

The microscopic scores were obtained by two different pathologists blinded to evaluation. Histopathological injury scores included inflammatory infiltrate and tissue damage (Schmidt et al., 2010). Points for infiltration were given as follows: 0, no infiltration; 1, increased number of inflammatory cells in the lamina propria; 2, inflammatory cells extending into the submucosa; and 3, transmural inflammatory infiltrates; and, for tissue damage: 0, no mucosal damage; 1, discrete epithelial lesions; 2, erosions or focal ulcerations; and 3, severe mucosal damage with extensive ulceration extending into the bowel wall.

### Enzyme-linked Immunosorbent Assay

The colon tissue of the mice was collected, and RIPA (Radio Immunoprecipitation Assay) buffer was used to lyse tissue at a ratio of 1:10, homogenized with an electric homogenizer in ice water, incubated at 4°C for 30 min, and centrifugated at 12,000 rpm for 10 min, and the tissue supernatant was obtained and analyzed. The total protein in each mouse was quantified with a total protein detection kit (Aidlab Biotechnologies Co., Ltd., Beijing, China). Where indicated, the samples were normalized to 5000 ng/mL in colon tissue supernatants of the respective experiment. The levels of cytokines, IL-7, IL-15, and IL-21, were measured using commercial enzyme-linked immunosorbent assay (ELISA) kits (BD Biosciences, NJ, United States) following the manufacturer's protocol, and optical density values of cytokines in each sample were detected on a microplate reader (Thermo, Varioskan, MA, United States). Then, each cytokine was quantified basally based on a standard curve established using an ELISA kit.

### Flow Cytometry

The numbers of CD45RA^+^CD62L^+^CCR7^+^ (naïve T) cells, CD45RA^−^CD62L^+^CCR7^+^ (TCM) cells, and CD45RA^−^CD62L^−^CCR7^+^ (TEM) cells in peripheral blood were detected by flow cytometry, and their CD4^+^ and CD8^+^ subsets were assessed too. The obtained lymphocytes were incubated with fluorescence-conjugated monoclonal antibodies in a staining buffer. Eight-color flow cytometry analysis (*n* = 8) was performed on a FACSCalibur device (Becton-Dickinson, CA, USA). A lymphocyte suspension was prepared as follows: 100 μL of anticoagulant, 100 μL of RPMI 1640 medium, and 1 mL of hemolysin were added to fresh blood and incubated for 15 min. The mixture was centrifuged at 300 g for 5 min, and the supernatant was removed. Then, 1 mL of stain buffer was added, and the cells were rinsed twice and centrifuged at 350 g for 5 min. The supernatant was discarded, and the cells were resuspended in 100 μL stain buffer. Further, 1 μg FcR blocking sealant was added and incubated at 4°C for 8 min. Primary antibodies were added and incubated for 15 min at room temperature. The cells were washed twice with 1 mL of stain buffer, the supernatant was discarded, and 500 μL of stain buffer was added to resuspend cells. The samples were detected using a FACSCanto II flow cytometer (BD Biosciences, NJ, United States). The following mAbs were used: PE rat anti-mouse CD4 (1:200), Alexa Fluor 647 rat anti-mouse CCR7 (1:100), BV510 rat anti-mouse CD45RA (1:100), BV421 rat anti-mouse CD62L (1:100), and FITC rat anti-mouse CD8 (1:100) (BD Biosciences). The limits for the quadrant markers were set based on negative populations and isotype controls. The analysis of acquired data was performed with the FlowJo software (Tree Star, OR, United States).

### Western Blotting

The normalized supernatants (5 μg/μL) of colonic tissues were prepared as described in *Enzyme-linked Immunosorbent Assay*. An equivalent amount of protein in each sample was fractionated onto sodium dodecyl sulfate–polyacrylamide gel electrophoresis and transferred to a polyvinylidene fluoride membrane (PVDF) with a Bio-Rad Western blot apparatus. The PVDF membranes were blocked with 5% fat-free milk or 5% bovine serum albumin and then incubated overnight with the following primary antibodies at 4°C. The primary antibodies were JAK1 (1:2000), STAT5 (1:1000), p-STAT5 (1:800), PIAS1 (1:1000), and GAPDH (1:5000) (Abcam, MA, USA). These membranes were treated with the corresponding secondary antibody [HRP-conjugated AffiniPure Goat Anti-Rabbit IgG or HRP-conjugated AffiniPure Goat Anti-Mouse IgG (1:5000–1:10000) (Proteintech, IL, United States] for 1-2 h at room temperature. Subsequently, these membranes were visualized with ECL western blot substrate. The specific protein bands were scanned with a UVP Chen Studio (Analytik Jena, Germany) and quantified using Image-Pro Plus 6.0 software (Media Cybernetic, MD, United States).

### Statistical Analysis

Data were expressed as the mean ± standard error of mean (SEM). Statistical analyses were carried out using GraphPad Prism 8.0 software (CA, United States). Student *t* test or one-way analysis of variance (ANOVA), followed by the Tukey test for multiple comparisons, was performed to determine significance. All *P* values less than 0.05 indicated a statistically significant difference.

## Results

### Curcumin Relieved DSS-Induced Colitis

**FIGURE 1:**
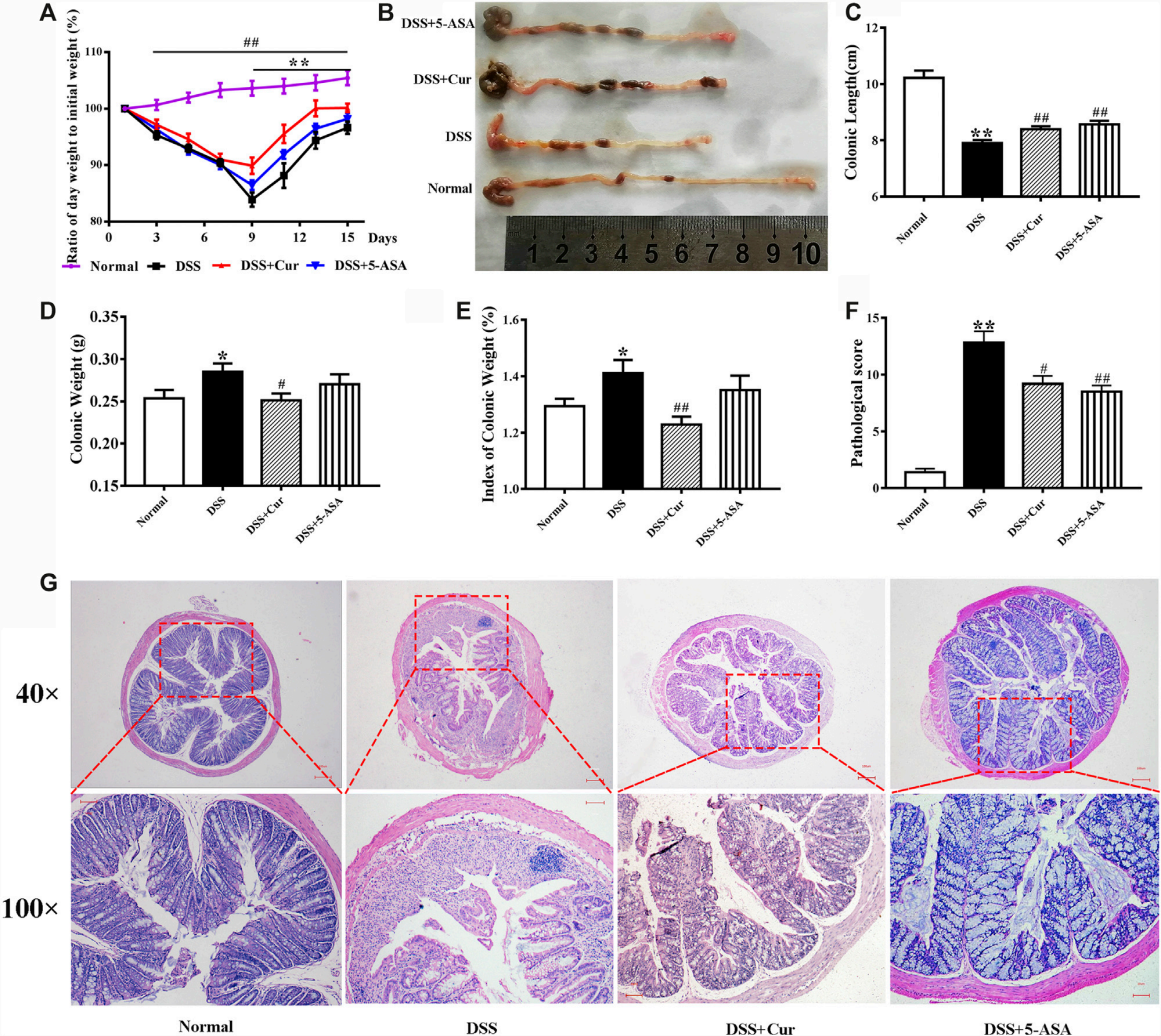
Therapeutic evaluation of curcumin on DSS-induced colitis in mice. **(A)** Ratio of day weight to initial body weight. **(B)** Changes in colonic length by naked eye. **(C)** Colonic length. **(D)** Colonic weight. **(E)** Index of colonic weight. **(F)** Histopathological score. **(G)** Histological appearance of the colons from individual groups of mice; the colonic sections were stained with hematoxylin and eosin. Data are representative images or as the mean ± SEM (*n* = 10 mice per group). ^*^ *P* < 0.05 and ^**^ *P* < 0.01 versus the DSS group, ^**#**^ *P* < 0.05 and ^**##**^ *P* < 0.01 versus the normal group, analyzed by ANOVA or Student *t* test.

DSS-induced experimental colitis is a classic model for studying the pathogenesis of IBD and the techniques and drug development for IBD treatment. In the present study, hematochezia, diarrhea, and rough and lusterless hair of BALB/c mice were visible with naked eyes after 4–5 days of DSS induction. The mice in the DSS group had significantly shorter colonic length (Figure 1B,C), higher colonic weight (Figure 1D) and colonic weight index (Figure 1E), and lower body weight change rate (Figure 1A), compared with the mice in the normal group. Pathological observations found disorganized mucosal structure, thickened colonic wall, ulcer formation, severe congestion, and edema, as well as inflammatory cell infiltration, in mice with colitis in the DSS group (Figure 1G). In addition, colonic histopathological damage scores were higher in the DSS group than in the normal group (Figure 1F). This was consistent with previous reports (Ge et al., 2020), suggesting that the DSS-induced experimental colitis was successfully replicated.

After curcumin treatment, the mice in the DSS + Cur group had significantly higher colonic length (Figure 1B,C) and significantly lower colonic weight (Figure 1D) and colonic weight index (Figure 1E) compared with mice in the DSS group. The ratio of daily body weight to initial body weight of mice was significantly higher in the DSS + Cur group than in the DSS group from day 9 (two doses) to the end of the experiment (Figure 1A). The mice in the DSS + Cur and DSS + 5-ASA groups showed a significant improvement in pathological injury, suppression of ulceration and epithelial proliferation, less inflammatory cell infiltration (Figure 1G), and significantly lower pathological injury scores compared with mice in the DSS group (Figure 1F). These results suggested that curcumin effectively relieved UC in mice.

### Curcumin Regulated the Expression of Cytokines in Colon Tissue

**FIGURE 2:**
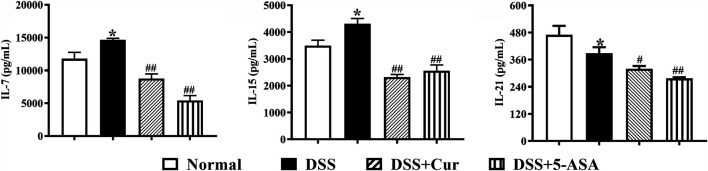
Curcumin regulated the expression levels of inflammatory cytokines IL-7, IL-15, and IL-21 in mice with colitis. The levels of cytokines, **(A)** IL-7, **(B)** IL-15, and **(C)** IL-21, were measured by ELISA. Data are presented as mean ± SEM (*n* = 8). ^**#**^ *P* < 0.05 and ^**##**^ *P* < 0.01 versus the normal group, ^*^ *P* < 0.05 and ^**^ *P* < 0.01 versus the DSS group, analyzed by ANOVA or Student *t* test.

Cytokines play an important role in intestinal homeostasis and inflammation-related pathological processes (Friedrich et al., 2019), and their abnormal expression is typical for UC, such as IL-7, IL-15, and IL-21. The levels of IL-7 (Figure 2A) and IL-15 (Figure 2B) in the colon tissue were significantly increased, while the levels of IL-21 (Figure 2C) significantly decreased in the DSS group compared with the normal group. After 7 days of treatment with curcumin and melaxazine, the levels of IL-7 (Figure 2A), IL-15 (Figure 2B), and IL-21 (Figure 2C) in the colon tissues were significantly lower in the DSS + Cur and DSS + 5-ASA groups than in the DSS group. These results suggested that curcumin effectively inhibited the secretion of proinflammatory cytokine in mice with colitis.

### Curcumin Regulated Memory T-Cell Differentiation

**FIGURE 3:**
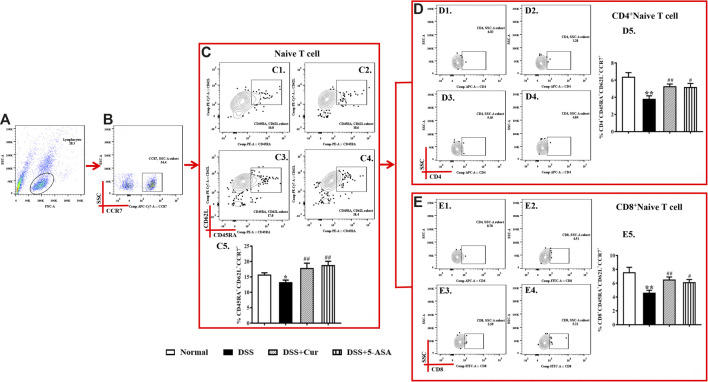
Curcumin regulated the number of naïve T cells in colitis mice. **(A)** Total lymphocytes in peripheral blood. **(B)** CCR7+ lymphocytes measured by flow cytometry. **(C)** Double-positive CD45R and CD62L lymphocytes; the cells in the upper left corner are naïve cells; C1–C4 represent naïve T cells in the normal, DSS, DSS + Cur, and DSS + 5-ASA group in order; C5: statistical analysis of naïve T cell frequencies in these four groups. **(D)** Flow cytometry analysis of CD4^+^ naïve T cell frequencies; D1–D4 represent naïve T cells in the normal, DSS, DSS + Cur, and DSS + 5-ASA groups in order; D5: statistical analysis of CD4^+^ naïve T cells frequencies in these 4 groups. **(E)** Frequency of CD8^+^ naïve T cells analyzed by flow cytometry. E1–E4 represent naïve T cells in the normal, DSS, DSS + Cur, and DSS + 5-ASA groups in order; E5: statistical analysis of CD4^+^ naïve T cells frequencies in these four groups. Data are presented as mean ± SEM (*n* = 10). ^**#**^ *P* < 0.05, ^##^ *P* < 0.01 versus the normal group; ^*^ *P* < 0.05 and ^**^ *P* < 0.01 versus the DSS group.

When T cells are not stimulated with antigens, naïve T cells are in a quiescent state and biologically marked by triple positivity for lymph node homing receptor CD62L, chemokine receptor 7 CCR7, and leukocyte common antigen CD45RA (Figure 3A–C). In this study, CD45RA^+^CD62L^+^CCR7^+^ cells were naïve T cells (Figures 3C1–4). The frequency of CD45RA^+^CD62L^+^CCR7^+^cells was significantly lower in the DSS group than in the normal group (Figures 3C1–5). This finding indicated that DSS antigen activated naïve T cells to differentiate into effector T cells. The frequency of CD45RA^+^CD62L^+^CCR7^+^ cells significantly increased in the DSS + Cur and DSS + 5-ASA groups compared with the DSS group (Figures 3C1–5). The frequency of two subsets of naïve T cells, CD4^+^ naïve T cells (Figures 3D1–5) and CD8^+^ naïve T cells (Figures 3E1–5) was significantly lower in the DSS group than in the normal, DSS + Cur, and DSS + 5-ASA groups. These results suggested that curcumin could effectively promote the number of naïve T cells and their subsets in mice with colitis.

**FIGURE 4:**
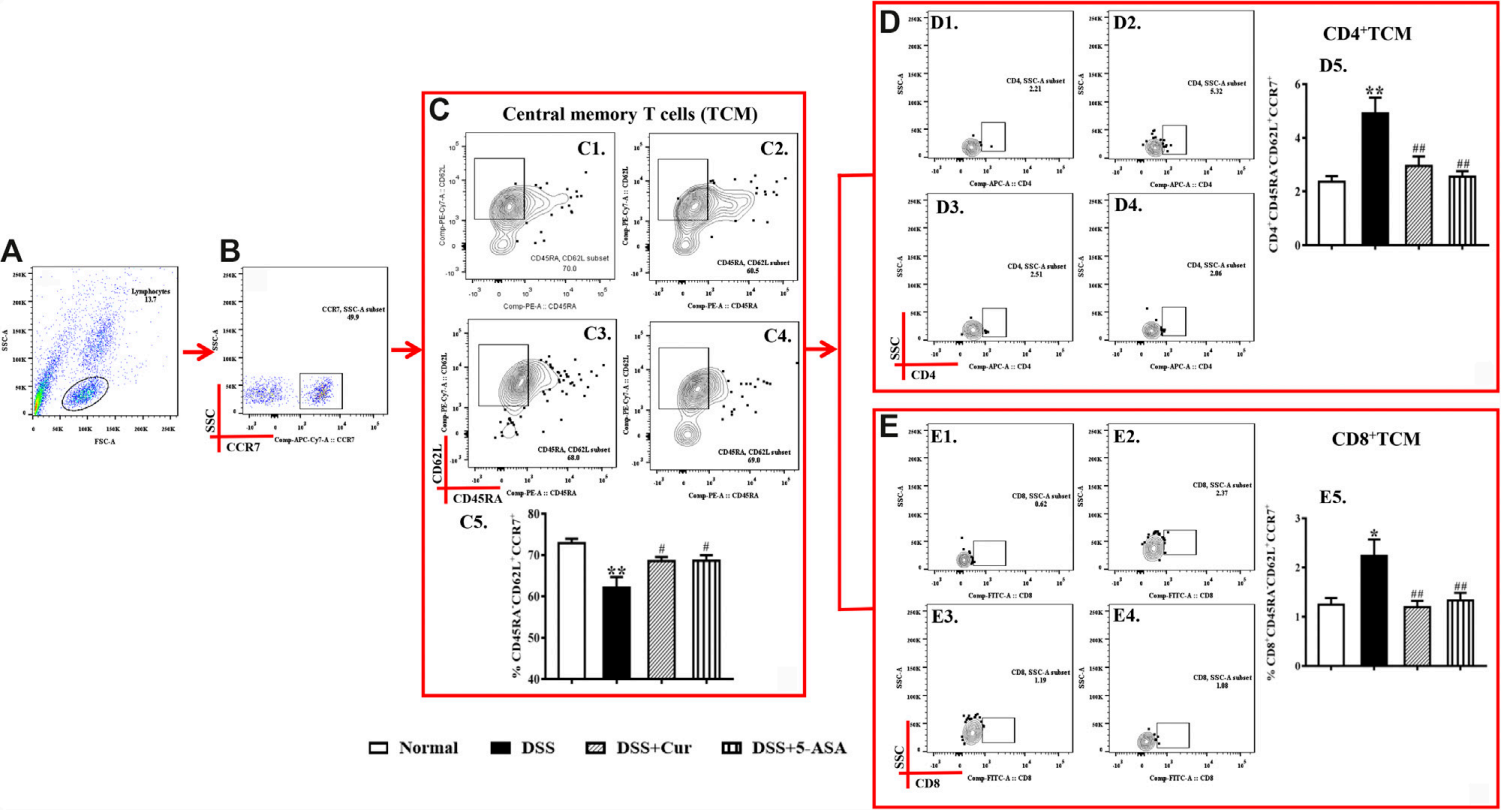
Curcumin regulated TCM cells in mice with colitis. (A) Total lymphocytes in peripheral blood. **(B)** CCR7+ lymphocytes measured by flow cytometry. **(C)** The CD45RA−CD62L+ labelled cells are TCM cells, located in the upper right corner; C1–C4 represent naïve T cells in the normal, DSS, DSS + Cur, and DSS + 5-ASA groups in order; C5: statistical analysis of TCM cells frequencies in these four groups. **(D)** Flow cytometry analysis of CD4^+^ TCM cell frequencies; D1–D4 represent CD4^+^ TCM cells in the normal, DSS, DSS + Cur, and DSS + 5-ASA groups in order; D5: statistical analysis of CD4^+^ TCM cell frequencies in these 4 groups. **(E)** The frequency of CD8^+^ TCM cells distribution analyzed by flow cytometry; E1–E4 represented CD8^+^ TCM cells in the normal, DSS, DSS + Cur, and DSS + 5-ASA groups in order; E5: statistical analysis of CD8^+^ TCM cells frequencies in these four groups. Data are presented as mean ± SEM (*n* = 10). ^#^ *P* < 0.05 and ^**##**^ *P* < 0.01 versus the normal group; ^*^ *P* < 0.05 and ^**^ *P* < 0.01 versus the DSS group.

Upon antigen activation, naïve T cells give rise to long-term memory T cells, which are T cells that could proliferate clonally through the lymphatic circulation back to secondary lymphoid organs and differentiate into TEM cells upon restimulation with homologous antigens. In this study, CD45RA^−^CD62L^+^CCR7^+^ cells were TCM cells (Figure 4C1–4). The number of CD45RA^−^CD62L^+^CCR7^+^ cells significantly reduced in mice with colitis in the DSS group compared with the normal group (Figures 4C1–5), but the numbers of two main subsets of TCM cells, CD4^+^ TCM (Figures 4D1–5) and CD8^+^ TCM (Figures 4E1–5), were significantly higher in the DSS group than in the normal group. After the mice with colitis were treated with curcumin and mesalazine, the number of TEM cells (Figures 4C1–5) was significantly higher than that in mice with untreated colitis, while the number of CD4^+^ TEM (Figures 4D1–5) and CD8^+^ TEM cells (Figures 4E1–5) decreased significantly. This study showed that curcumin could regulate the differentiation of TCM cells and their subpopulations.

**FIGURE 5:**
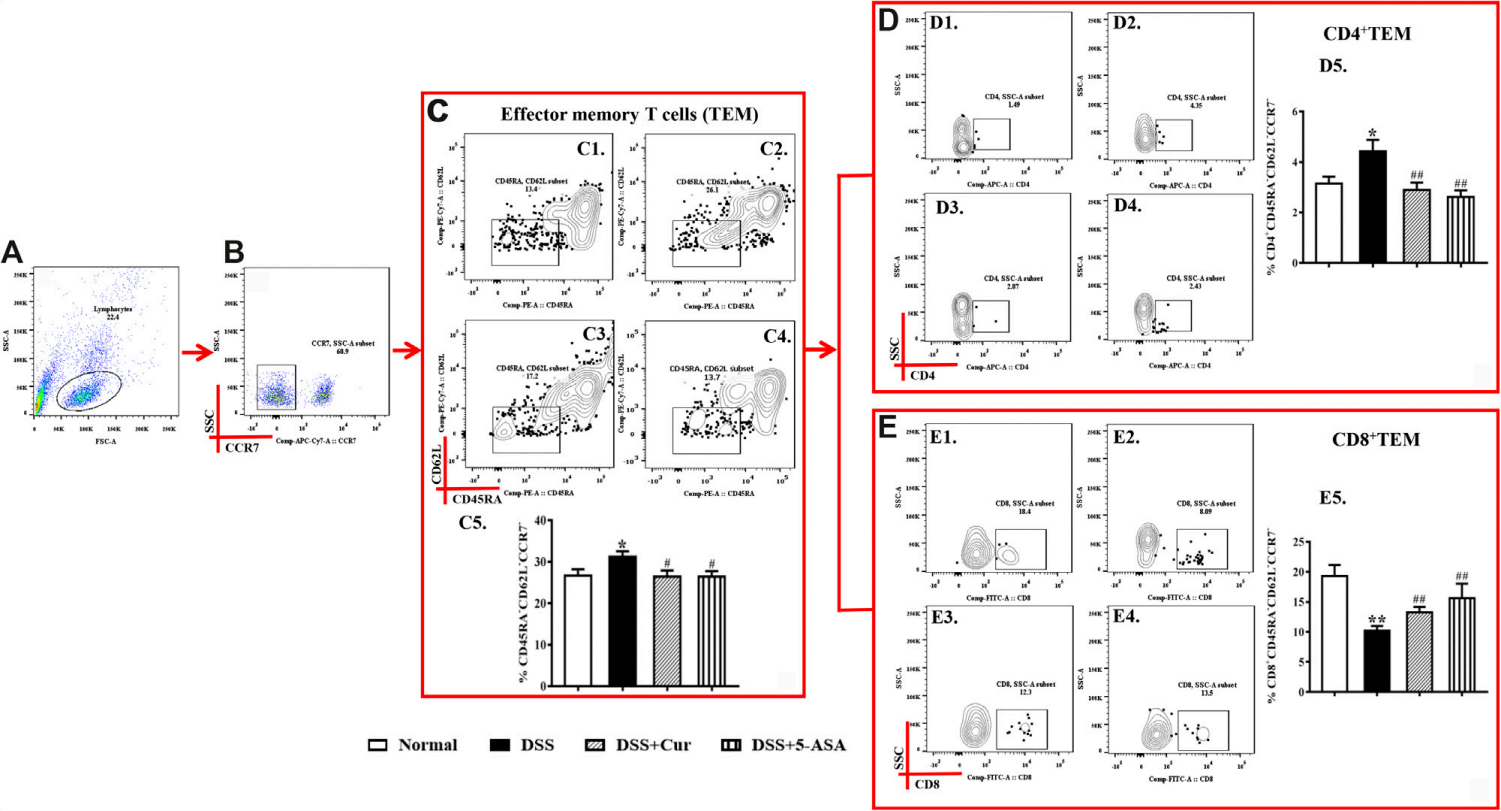
Curcumin regulated TEM cells in mice with colitis. **(A)** Total lymphocytes in peripheral blood. **(B)** CCR7-lymphocytes measured by flow cytometry. **(C)** The CD45RA−CD62L− labelled cells are TCM cells, located in the lower right corner; C1–C4 represent TEM cells of the normal, DSS, DSS + Cur, and DSS + 5-ASA groups in order; C5: statistical analysis of TEM cells frequencies in these four groups. **(D)** Flow cytometry analysis of CD4+ TEM cells frequencies; D1–D4 represent CD4+ TEM cells in the normal, DSS, DSS + Cur, and DSS + 5-ASA groups in order; D5: statistical analysis of CD4+ TEM cells frequencies in these 4 groups. **(E)** The frequency of CD8+ TEM cells distribution analyzed by flow cytometry; E1–E4 represented CD8+ TEM cells in the normal, DSS, DSS + Cur, and DSS + 5-ASA groups in order; E5: statistical analysis of CD8+ TEM cells frequencies in these four groups. Data are presented as mean ± SEM (n = 10). ^#^P < 0.05 and ^##^P < 0.01 versus the normal group; ^*^ *P* < 0.05 and ^**^ *P* < 0.01 versus the DSS group.

TEM cells can migrate to peripheral inflammation sites and exert an immune function; they do not express CCR7, CD45RA, and CD62L on their surface. In this study, CD45RA^−^CD62L^−^CCR7^-^ cells were TEM cells. The number of TEM cells (Figure 5C1–5) in the peripheral blood of mice in the DSS group was significantly higher than that in the normal group, the number of their subsets CD4^+^ TEM (Figure 5D1–5) significantly increased, and CD8^+^ TEM (Figure 5E1–5) remarkably reduced. The numbers of TEM cells (Figure 5C1–5) and their subsets CD4^+^ TEM (Figure 5D1–5) significantly reduced in the peripheral blood after 7 days of treatment with curcumin and 5-ASA compared with DSS treatment, and CD8^+^ TEM cells remarkably upregulated (Figure 5E1–5). The results showed that curcumin could effectively regulate the differentiation balance of TEM cells in DSS-induced experimental colitis.

### Curcumin Regulated JAK1/STAT5 Signal Activation

**FIGURE 6:**
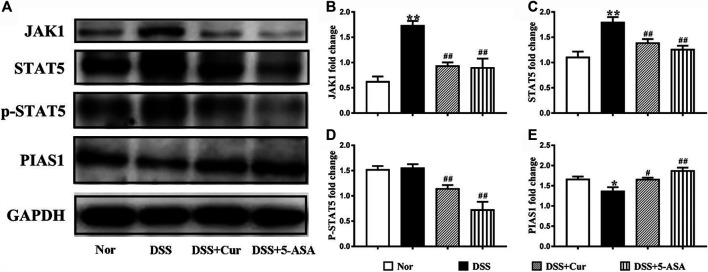
Curcumin regulated JAK1/STAT5 signaling activation in mice with colitis. **(A)** The protein expression in the JAK/STAT5 signaling pathway, such as JAK1, STAT5, p-STAT5 and PIAS, was analyzed by western blotting analysis. **(B)** Quantitative analysis of the expression of JAK1 protein. **(C)** Quantitative analysis of the expression of STAT5 protein. **(D)** Quantitative analysis of the expression of p-STAT5 protein. **(E)** Quantitative analysis of the expression of PIAS1 protein. Data are presented as mean ± SEM (*n* = 8). ^**#**^ *P* < 0.05 and ^**##**^ *P* < 0.01 versus the normal group, ^*^ *P* < 0.05 and ^**^ *P* < 0.01 versus the DSS group, analyzed by ANOVA or Student *t*-test.

JAK1/STAT5 signaling is activated with the involvement of γc family receptors to regulate memory T-cell differentiation (Rochman et al., 2009). Therefore, the western blotting technique was further used to measure the protein levels of JAK1, STAT5, p-STAT5, and JAK1/STAT5 signal negative regulator PIAS1 (protein inhibitor of activated signal transducer and activators of transcription 1) in colon tissue (Figure 6A). The levels of JAK1 (Figure 6B) and STAT5 (Figure 6C) proteins in the colon tissues of mice significantly increased, the changes in the levels of p-STAT5 (Figure 6D) were not statistically significant, and the protein level of PIAS1 (Figure 6E) significantly decreased in the DSS group compared with the normal group. The colonic protein levels of JAK1 (Figure 6B), STAT5 (Figure 6C), and p-STAT5 (Figure 6D) were significantly decreased, and the protein level of PIAS1 (Figure 6E) was significantly increased in the DSS + Cur and DSS + 5-ASA groups compared with the DSS group. The results indicated that curcumin could effectively inhibit the activation of the JAK1/STAT5 signaling pathway in DSS-induced experimental colitis.

## Discussion

In the present study, the onset of IBD in humans was simulated using an experimental DSS-induced UC. After DSS induction, the mice with colitis showed significant weight loss, hematochezia, and an increase in disease activity index score; colonic shortening, epithelial erosion of the colonic mucosa on microscopic examination, formation of ulcers and granulation tissue, disorganized crypt structure, and inflammatory cell infiltration; and a significant increase in the levels of proinflammatory cytokines IL-7, IL-15, and IL-21 in colonic tissues. These results suggested that DSS successfully recapitulated colitis. The number of naïve T cells CD4^+^, naïve T and CD8^+^ naïve T cells decreased significantly; the number of TCM cells decreased significantly, while the number of CD4^+^ TCM and CD8^+^ TCM cells increased significantly; and the number of TEM and CD4^+^ TEM increased significantly, while CD8^+^ TEM cells decreased significantly. These results indicated that the numbers of memory T cells and their subsets significantly changed in DSS-induced colitis, suggesting that immune memory cell differentiation disorders might be closely related to the onset and development of IBD.

In terms of acquired immunity, "memory" is now generally considered to be the ability to respond once to an antigen and a stronger response the next time the same antigen is stimulated (Netea et al., 2016). The main antigens in IBD are intestinal commensal and autoantigens, which are not completely eliminated resulting in recurrent episodes of IBD (Franzosa et al., 2019). Given the aging and atrophy of the human thymus, naïve T cells, which are not readily available in the thymus, continue to differentiate into colon effector T cells (Kondo et al., 2019). Naïve T cells differentiate rapidly into effector T cells under the stimulation by TCRs and costimulatory molecules (Jenkins et al., 2001). When antigen elimination is cleared, apoptosis of most effector T cells occurs and the remaining effector T cells are converted into long-lived memory T cells (Pepper and Jenkins, 2011). Thus, when the physical barrier of the intestinal mucosa is compromised, symbiotic bacteria enter the lamina propria through the leaky gut to induce an inflammatory response (Friedrich et al., 2019). TCM cells, which express CCR7 and CD62L, migrate through the bone marrow and bloodstream to secondary lymphoid organs (Reinhardt et al., 2001), proliferate, and differentiate into TEM cells, which then exert their effects at the site of lost inflammation. When the intestinal tract is in direct contact with the outside world and the intestinal mucosal epithelium is inevitably damaged, intestinal commensal bacteria and auto-reactive antigens enter the mucosal tissues and repeatedly stimulate them, leading to abnormal activation of memory T cells. Therefore, the dynamic equilibrium of pathogenic memory T cells plays an important role in the pathogenesis of autoimmune diseases.

The immune memory dysfunction is the key characteristic of IBD. Growing evidence shows that IBD is caused by innate and adaptive immune system responses to intestinal symbionts, which frequently recur and persist despite going into remission after treatment (Weingarden and Vaughn, 2017; Nishida et al., 2018). In mice with UC, excessive inflammatory CD4^+^ TEM cells infiltrated into the epithelium and lamina propria of colon mucosa (Wirtz et al., 2017). CD4^+^ Tm (CD4^+^ CD45RB^high^) cells isolated from the lamina propria of mice with colitis were cultured *in vitro* for 8 weeks and transplanted into SCID mice to cause acute colitis, and CD4^+^CD44^+^CD62L^−^ memory T cells were found to overaggregate in the lamina propria of the colon (Takahara et al., 2013). The differentiation status of CD4^+^ Tm in the gut determines the relapse-remitting process of chronic UC (Jameson and Masopust, 2018). These studies suggested that targeting colon-causing inflammatory memory T cells would be one of the targets of IBD therapy. Japanese scholars, Fujii et al. (2006), effectively alleviated IBD by interfering with memory T-cell differentiation homeostasis. CD4^+^CD45RB^high^ T cells were injected into SCID mice to induce immune memory dysfunction in colitis mice. A 2 week intervention treatment with the immunosuppressant fingolimod (FTY20) resulted in significant remission of colitis. The levels of effector memory T cells, such as CD4^+^CD62L^−^ (CD4^+^TEM) T cells, and the expression of the homing cell adhesion molecule CD44 in the peripheral blood, spleen, lamina propria of the small intestine, and mesenteric lymph nodes of mice with colitis were found to be decreased.

Curcumin is a natural hydrophobic polyphenol extracted from turmeric rhizome, which has been widely used as an herbal medicine in China and Southeast Asia for hundreds of years (Lestari and Indrayanto, 2014; He et al., 2015). It has many pharmacological activities, such as anti-inflammatory, antioxidant, and antitumor (Sharma et al., 2005). Curcumin is widely used in the clinical treatment of human UC patients and the evaluation of animal therapeutic effect of colitis (Epstein et al., 2010; Sareen et al., 2013). In this study, colonic injury was alleviated or disappeared, including a decrease in colonic weight index, downregulation of histopathological scores, and restoration of colonic length, and the numbers of naïve, TEM, and TCM cells and their respective subpopulations were restored to normal after 14 days of continuous treatment with curcumin in mice with DSS-induced colitis. Strong evidence showed that curcumin might treat IBD by modulating memory T-cell differentiation.

In our study, DSS-induced mice treated with curcumin showed significant decreases in the levels of proinflammatory cytokines IL-7, IL-15, and IL-21 in colonic tissues. Cytokines IL-7, IL-15, and IL-21 mediate transmission signals following binding of the common γ-chain (Kohn et al., 2014) (JAK1 and γc) of type I receptors with JAK1 and JAK3 (Rochman et al., 2009; Salas et al., 2020), resulting in phosphorylation and nuclear translocation of STAT5A and/or STAT5B (in response to IL-2). Molecular interactions between JAKs and STATs mediate cellular responses that play a fundamental role in both intestinal homeostasis and inflammation (Coskun et al., 2013). Molecules that interfere with these interactions, especially those targeting JAK1 and JAK3, such as TNF antagonists (Jones-Hall and Nakatsu, 2016), have shown promising efficacy and safety in IBD treatment. The protein levels of JAK1, JAK3, and STAT5 were downregulated in mice with colitis after curcumin treatment, indicating that curcumin inhibited JAK-STAT signal activation. This finding suggested that curcumin might interfere with JAK-STAT signaling to modulate the differentiation of memory T cells for UC, which requires further exploration with a separate JAK-STAT intervention group or specific gene knockout mice.

In addition, cytokines IL-7, IL-15, and IL-21 play key roles in the differentiation of memory T cells. L-7 receptors are highly expressed in memory T cells (Tm), naïve T cells (Tn) receptors. IL-7 activates IL-7 receptor α and induces proliferation and long-term survival of effector memory CD4^+^ T cells (Takahara et al., 2013), leading to chronic colitis. IL-15 regulates memory CD8^+^ T cell lifespan and effector function (Yoshihara et al., 2006). Under steady-state or lymphopenic conditions, IL-21 acts directly on CD8^+^ T cells, favoring the accumulation of TE/TEM populations (Tian et al., 2016). It is implied that regulation of memory T cell differentiation through intervention with IL-7, IL-15, and IL-21 is an effective measure for the treatment of ulcerative colitis. In the present study, the decreasing trend of IL-7 concentration in colitis mice after curcumin intervention was consistent with CD4^+^ TEM; however, the trend of IL-15 and IL-21 change was not consistent with CD8^+^ TEM cells, probably due to the fact that IL-15 and IL-21 were detected in colonic tissue while CD8^+^ TEM was in blood. Therefore, our next step is to further test the levels of cytokines IL-7, IL-15, and IL-21 by isolating CD4^+^ TEM, CD8^+^ TEM, and coculturing them with curcumin *in vitro*. Surprisingly, the levels of the proinflammatory cytokine IL-21 were lower in the colitis mice than in the normal group of mice. This may be due to the fact that the source of IL-21 is not only produced by activated CD4^+^ T cells (Harada et al., 2006), but also derived from other cells such as natural killer cells. We will explore the role played by IL-21 in DSS-induced colitis by knocking out the IL-21 gene.

## Data Availability Statement

The original contributions presented in the study are included in the article/Supplementary Material; further inquiries can be directed to the corresponding authors.

## Ethics Statement

The animal study was reviewed and approved by the Animal Care and Use Committee of Jiangxi University of Traditional Chinese Medicine.

## Author Contributions

D-YL and H-MZ conceived and designed the experiments. Y-BZ, Z-PK, B-GZ, JL, WZ, and H-YW performed the experiments. D-YL and H-MZ contributed reagents/materials/analytical tools. D-YL and Y-BZ analyzed the data. Y-BZ and D-YL wrote the manuscript.

## Funding

This study was supported in part by the National Natural Science Foundation of China (Nos. 82060799, 8180792, 81760838, and 81760808), Natural Science Foundation of Jiangxi Province (Nos. 20192ACB20015, 20192BAB215050, and 20181BAB205082), Education Department of Jiangxi Province (Nos. GJJ181582, GJJ196047, and 20181969), and 1050 Young talents project (No. 1141900603) and first-class subjects starting funds (Nos. JXSYLXK-ZHYI022, JXSYLXK-ZHYAO132, and JXSYLXK-ZHYAO108) of Jiangxi University of Traditional Chinese Medicine.

## Conflict of Interest

The authors declare that the research was conducted in the absence of any commercial or financial relationships that could be construed as a potential conflict of interest.
